# Interkingdom Communication via Extracellular Vesicles: Unraveling Plant and Pathogen Interactions and Its Potential for Next-Generation Crop Protection

**DOI:** 10.3390/microorganisms12122392

**Published:** 2024-11-22

**Authors:** Fei Li, Yuntong Lu, Kuanling Xi, Yuke Li, Xiaoyan Chen, Puchang Wang, Xiaolong Huang

**Affiliations:** 1 School of Life Sciences, Guizhou Normal University, Guiyang 550025, China; 18984028920@163.com (Y.L.); xikuanling@163.com (K.X.); yk_liyuke@sina.com (Y.L.); chenxy8387@126.com (X.C.); wangpuchang@163.com (P.W.); huangxiaolong@gznu.edu.cn (X.H.); 2 State Key Laboratory of Microbial Technology, Shandong University, Qingdao 266237, China

**Keywords:** extracellular vesicles (EVs), cross-kingdom communication, small regulatory RNAs (sRNAs), crop protection, gene silencing

## Abstract

Recent advancements in the field of plant–pathogen interactions have spotlighted the role of extracellular vesicles (EVs) as pivotal mediators of cross-kingdom communication, offering new vistas for enhancing crop protection strategies. EVs are instrumental in the transport of small regulatory RNAs (sRNAs) and other bioactive molecules across species boundaries, thus playing a critical role in the molecular warfare between plants and pathogens. This review elucidates the sophisticated mechanisms by which plants utilize EVs to dispatch sRNAs that silence pathogenic genes, fortifying defenses against microbial threats. Highlighting both eukaryotic and prokaryotic systems, this review delves into the biogenesis, isolation, and functional roles of EVs, illustrating their importance not only in fundamental biological processes but also in potential therapeutic applications. Recent studies have illuminated the significant role of EVs in facilitating communication between plants and pathogens, highlighting their potential in host-defense mechanisms. However, despite these advancements, challenges remain in the efficient isolation and characterization of plant-derived EVs. Overcoming these challenges is critical for fully harnessing their potential in developing next-generation crop protection strategies. This review proposes innovative strategies for utilizing RNA-based interventions delivered via EVs to bolster plant resilience against diseases. By integrating the latest scientific findings with practical applications in agriculture, this review aims to enhance the connection between fundamental plant biology and the development of innovative crop management technologies.

## 1. Introduction

Agricultural productivity and the stability of ecosystems are substantially compromised by plant pathogens. Understanding the complex biological interplay between plants and these harmful agents is crucial for developing innovative, effective disease management strategies applicable to both agricultural and natural environments. Central to this interplay is the intricate molecular communication involving the exchange of effector molecules such as proteins and toxins from pathogens to plant hosts, and defensive antimicrobial compounds from plants to their adversaries [1,2,3]. Recent research has underscored the importance of RNA molecules, particularly regulatory small RNAs (sRNAs), which traverse interspecies boundaries to enact gene silencing, playing a pivotal role in the dynamics of plant–pathogen interactions [4,5,6,7,8,9]. These sRNAs are produced by Dicer or Dicer-like enzymes in plants and are linked with Argonaute proteins, targeting and silencing complementary genes in pathogens, thus forming a vital line of defense. Additionally, extracellular vesicles (EVs) are critical for ferrying these sRNAs, mediating this inter-organismal molecular exchange and significantly affecting the dynamics of host–pathogen interactions [2,10].

In both plant and animal systems, EVs are recognized increasingly for their essential roles in cellular communication, facilitated by their capacity to transport various biological molecules such as proteins, lipids, and nucleic acids. EVs, isolated from different biological fluids or cultured media, are typically characterized by their distinct biophysical properties, markers, and functions depending on their cellular origin [11]. These vesicles are broadly categorized into exosomes, microvesicles (also known as shedding vesicles), and apoptotic bodies. Exosomes are tiny vesicles originating from the internal budding of multivesicular bodies (MVBs), released into the extracellular space when MVBs merge with the plasma membrane. This process allows the selective export of cargoes meant for distant targets [12]. Microvesicles are larger, formed at the plasma membrane through outward budding and fission [13]. This process encapsulates cytosolic contents reflecting the cell’s state during vesicle formation. Apoptotic bodies, formed during apoptosis, contain cellular debris and various bioactive molecules, aiding in the clearance of dying cells and preventing inflammation.

EVs are of great interest as vehicles for drug targeting and in fundamental biological research, but in vitro culture of animal cells usually achieves only small yields [14]. The exploration of other biological kingdoms promises comprehensive knowledge on EVs broadening the opportunities for basic understanding and therapeutic use [15,16,17]. Thus, plants might be sustainable biofactories producing nontoxic and highly specific nanovectors [18], whereas bacterial and fungal EVs are promising vaccines for the prevention of infectious diseases [19]. Importantly, EVs from different eukaryotic and prokaryotic kingdoms are involved in many processes including host–pathogen interactions, spreading of resistances, and plant diseases. More extensive knowledge of inter-species and interkingdom regulation could provide advantages for preventing and treating pests and pathogens. In this review, we present a comprehensive overview of EVs derived from eukaryota and prokaryota and we discuss how better understanding of their intercommunication role provides opportunities for both fundamental and applied biology.

The diverse origins and biogenesis pathways of EVs underscore their intricate roles in health and disease, serving as conduits for molecular signals across cellular boundaries. Initially identified in mammalian cells, the role of exosomes has broadened significantly, indicating their involvement in various biological processes and potential therapeutic and diagnostic applications. These vesicles encapsulate a variety of bioactive molecules, playing a crucial role in cellular communication across different biological systems—from fertilization and immune modulation to disease progression and tissue regeneration.

Despite considerable progress in understanding EVs in animal systems, the exploration of these vesicles in the plant kingdom and their interactions with microbial pathogens has been less rapid. The presence of plant-derived vesicles at sites of bacterial and fungal interactions has been confirmed through transmission electron microscopy [20]. However, challenges in EV isolation and detection have hindered a deeper understanding of their function in plant systems and interactions with phytopathogens such as bacteria, fungi, and oomycetes. Recent studies, however, have shed light on the significant role of EVs in facilitating communication between plants and pathogens, underscoring their potential in host-defense mechanisms [1,21,22,23,24]. This review synthesizes the latest findings on EVs derived from both plant cells and microbial sources, focusing particularly on their involvement in plant–pathogen interactions. We explore the complexities of EV biogenesis and secretion, examine the molecular details, and discuss the experimental challenges that need addressing to advance our comprehension of their biological roles. Additionally, the potential application of EVs in agriculture, particularly for delivering RNA molecules that protect crops, is briefly discussed, highlighting their prospective utility in enhancing crop resilience.

## 2. Plant Extracellular Vesicles

Plant extracellular vesicles (EVs) were first documented in carrot cell cultures using electron microscopy in 1967. Since their initial observation, plant EVs have been identified in a variety of plant fluids, including those from leaves, roots, and seeds undergoing hydration, as well as in the media used for pollen germination and growth [18,25,26,27,28]. Transmission and confocal microscopy have demonstrated that these vesicles play a crucial role in plant immune responses, often increasing in number during pathogenic attacks [20,23], which also leads to more frequent occurrences of multivesicular body (MVB) and plasma membrane fusion events.

Recent studies have expanded the understanding of the properties and biological implications of plant-derived EVs. These vesicles have been shown to carry distinct biomarkers and a diverse array of molecules including proteins, RNAs, and metabolites, suggesting their significant role in modulating physiological responses in recipient cells and the broader ecological interactions with other organisms [25,29,30]. Notably, plant EVs have been observed facilitating communication between plants and symbiotic fungi, further highlighting their role in cross-kingdom interactions [31].

In animal systems, a distinct subset of proteins, prominently including tetraspanins like CD9, CD63, CD37, CD81, and CD82, has been identified through proteomic analyses as highly concentrated within exosomes, serving as standard biomarkers for these vesicles. In contrast, the plant *Arabidopsis* harbors 17 TETRASPANIN (TET) family members, which, despite having low sequence similarity to their animal counterparts, share the characteristic transmembrane topology of tetraspanin proteins [32,33,34,35]. Notably, expression analyses have shown that TET8 and TET9 genes exhibit significant upregulation following infection by the fungal pathogen *Botrytis cinerea*, implicating them in plant defense mechanisms [20].

This leads to the use of “plant exosome” to denote tetraspanin-enriched vesicles emanating from MVBs into the extracellular plant space, or apoplast. In research on skeletal muscle myoblast cells, differential ultracentrifugation remains a cornerstone for EV isolation [16]: large EVs such as apoptotic bodies are typically isolated at lower speeds, medium-sized EVs like microvesicles at intermediate speeds, and smaller EVs including exosomes at very high speeds. Plant EVs show a similar enrichment for the TET8 marker in fractions obtained at approximately 100,000× *g* from leaf apoplastic fluids [36]. However, this method primarily enriches rather than purifies, as EVs of similar sizes may co-isolate. To refine the separation, high-speed density gradient ultracentrifugation is utilized, where EVs segregate into distinct density fractions, enabling more precise isolation of various vesicle subtypes [36]. For plants, this involves the use of gradients such as sucrose or OptiPrep, with TET8- and TET9-positive exosomes often concentrating in specific gradient fractions. The most sophisticated isolation technique employed is immunoaffinity isolation [32,36], which utilizes antibodies against specific EV markers to isolate subtypes cleanly, avoiding contamination from other cellular components. Notably, for plant systems, traditional GFP-tagging of TET proteins for immunoaffinity capture is unfeasible due to the internal positioning of their termini within the vesicles. Instead, antibodies recognizing the large extravesicular loop, specifically the EC2 domain of TET8, have been developed to enable effective isolation of TET8-positive exosomes.

In *Arabidopsis* Columbia-0, Penetration 1-positive extracellular vesicles (PEN1 EVs) have been isolated from leaf apoplastic fluid using a lower ultracentrifugation speed of 40,000× *g* [37]. Proteomic investigations of these vesicles reveal their content of proteins crucial for responding to biotic and abiotic stresses, prominently featuring the plant-specific protein Penetration 1 (PEN1), initially characterized as a syntaxin associated with the plasma membrane. The secretion of PEN1 is facilitated by an ADP ribosylation factor-GTP exchange factor (ARF-GEF), known as GNOM, which is implicated in the recycling endosome trafficking route, distinct from the multivesicular body (MVB) pathway used by other extracellular vesicles. This unique biogenesis pathway indicates that PEN1-positive EVs do not share the same biomarkers or origins as exosomes, which typically colocalize with *Arabidopsis* MVB-marker Rab5-like GTPase ARA6 within the plant cell. Instead, PEN1 EVs follow a different pathway, as evidenced by their lack of colocalization with ARA6-marked MVBs. Further differentiating them, PEN1-positive EVs are concentrated in a specific gradient density of 1.029 to 1.056 g/mL, distinct from the densities typical of TET-positive EVs [38,39].

Experimental observations using transgenic plants co-expressing fluorescently tagged fusion proteins, TET8-GFP and mCherry-PEN1, have also demonstrated that PEN1-positive EVs represent a separate class of vesicles within plant cells, distinct from those marked by TET8 [32]. These findings confirm the hypothesis that PEN1-positive and TET8-positive EVs are fundamentally different, likely involved in transporting diverse types of molecular cargos.

A distinct category of plant extracellular vesicles (EVs) originates from organelles known as exocyst-positive organelles (EXPOs). These were first identified in cells of *Arabidopsis* and tobacco (*Nicotiana tabacum*) that expressed a homolog of the exocyst component Exo70E2 [40]. EXPOs are characterized by a unique morphology that distinguishes them from multivesicular bodies (MVBs), and they operate independently of endosomes and autophagosomes. The role of AtExo70E2 in the formation of EXPOs is critical, yet the specific mechanisms underlying EXPO biogenesis remain largely undefined. Immunogold staining techniques have shown that EXPOs are spherical, double-membraned structures within cells. Upon fusion of their outer membrane with the plasma membrane, these organelles release single-membraned vesicles into the extracellular space [40]. Typically, the vesicles derived from EXPOs range between 200 and 500 nm in diameter, notably larger than typical exosomes, indicating that EXPOs may be a source of larger EVs in plants. In tobacco, it has been observed that the glycosyltransferases AtGALT14A, AtGALT29A, and AtGALT31A colocalize with the EXPO marker AtExo70E2 [41]. This colocalization suggests that EVs derived from EXPOs may play a role in the extracellular secretion of arabinogalactan proteins, which are essential components in plant biology [42].

## 3. Extracellular Vesicles in Mediating Small RNA Exchange and Plant Defense Mechanisms

The secretion of plant extracellular vesicles (EVs) is notably enhanced following pathogen infection, highlighting their critical function in plant defense strategies [1,20,21,23,43]. EVs serve as essential carriers for various biological materials, facilitating the transfer of crucial molecules such as RNAs, proteins, and metabolites between plants and their interacting microbial partners. This exchange mechanism underscores the evolutionary adaptation of both plants and microbes to leverage EVs as a means of communication and functional molecule exchange within cross-kingdom interactions.

### 3.1. Cross-Kingdom sRNA Trafficking via Plant Extracellular Vesicles

RNA interference (RNAi) is a universal gene-silencing tool across eukaryotes, crucial for controlling both innate and foreign gene activities. In plants, which do not have an immune system based on antibodies and lymphocytes like mammals, RNAi serves as a vital defense mechanism against a wide spectrum of pathogens including viruses, bacteria, fungi, and oomycetes [44,45,46,47]. This method has also been adapted through genetic engineering to enhance resistance to numerous agricultural threats.

**Figure 1:**
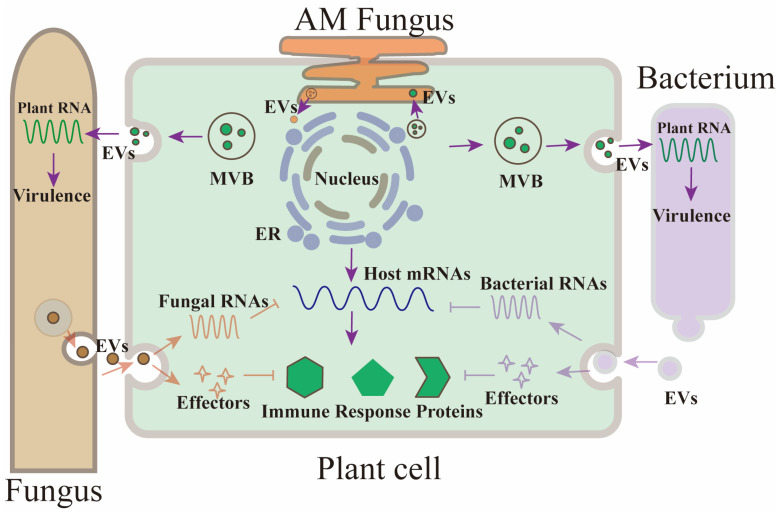
During pathogen invasion, plants release extracellular vesicles (EVs) into the extracellular space, containing small RNAs (sRNAs) that can be taken up by pathogens. These sRNAs specifically target and suppress pathogen virulence genes. Simultaneously, pathogens deploy sRNA effectors into host cells to suppress the plant’s immune responses. EVs have been identified at the plant-arbuscular mycorrhizal (AM) interface. Key abbreviations include AM (arbuscular mycorrhizal) and EV (extracellular vesicle).

Recent findings have illuminated the role of small RNAs (sRNAs) in bridging the communication between plant and pathogen, facilitating gene silencing across species barriers, a phenomenon known as cross-kingdom RNAi [9,20,48,49,50,51,52,53,54,55,56,57] (Table 1, Figure 1). Specifically, *Arabidopsis thaliana* transmits a specialized set of miRNAs and small interfering RNAs (siRNAs), including phased secondary siRNAs (phasiRNAs), to target and silence pathogenic genes in *B. cinerea*, a common fungal adversary [20]. These sRNAs are packaged into extracellular vesicles (EVs), particularly TET-positive exosomes, which are essential for their stability and functional delivery [32]. Studies show that these sRNAs are specifically enriched within certain gradient fractions where TET-positive exosomes are also concentrated. Treatment with ribonucleases can remove any sRNAs that may adhere externally to the vesicles or co-sediment unintentionally, confirming that the functional sRNAs are securely housed within the EVs [20]. Through the use of immunoaffinity capture, specifically tailored antibodies such as those targeting TET8, have enabled the selective isolation of these exosomes, further enriching our understanding of their role in sRNA transport [20,23,32,36]. Notably, *Arabidopsis* mutants lacking TET8 and TET9 exhibit reduced sRNA secretion and diminished ability to transport these molecules into fungal cells, underscoring the critical role of TET-positive exosomes in mediating cross-kingdom RNAi [20].

Extracellular vesicles (EVs) loaded with small RNAs (sRNAs) from plants are effectively absorbed by fungal cells [20] (Table 1, Figure 1). Research shows that a significant portion—over 70%—of plant sRNAs detected in *B. cinerea* protoplasts isolated from infected plants are also found within plant EVs, highlighting EV-mediated transport as a crucial pathway for delivering plant sRNAs. Double mutants of *tet8 tet9* exhibit increased vulnerability to fungal infections, and fungal cells from these mutants show a substantial reduction in the intake of plant sRNAs. These transported sRNAs specifically target fungal genes associated with virulence, evident from the reduced virulence of mutant fungal strains lacking these targeted genes compared to wild-type strains. Identification of such target genes in fungi, such as vacuolar protein sorting 51 (*Bc-Vps51*), the large subunit of the dynactin complex (*Bc-DCTN1*), and suppressors of actin-like phosphoinositide phosphatase—all crucial in vesicle trafficking and pathogenicity of *B. cinerea*—offers insights into potential virulence factors [20].This study underscores the critical role of EVs in facilitating sRNA movement into fungal cells, acting as a protective mechanism in cross-kingdom interactions. Further studies revealed that *Arabidopsis* also shuttles secondary phasiRNAs into oomycete pathogens like *Phytophthora capsici*, effectively silencing pathogen genes [58]. TET8 knockout mutants displayed significantly reduced levels of cellular glycosyl inositol phosphoceramides (GIPCs) and a decreased secretion of EVs, suggesting a link between TET8 and exosome production related to GIPCs [22]. This connection opens new avenues for exploring the role of TET proteins and TET-positive EVs in the transport of functional molecules. Recent findings also indicate that PEN1-positive EVs transport tiny RNAs (10–17-nt), though their biological functions remain to be clarified [59]. Additionally, similar sRNA-induced gene silencing mechanisms are observed in crop plants like cotton and wheat, where EV-delivered miRNAs target and diminish the virulence of fungal pathogens such as *Verticillium dahliae* [56,60] and *Fusarium graminearum* [61].

### 3.2. Plant RNA-Binding Proteins and Their Role in sRNA Loading into Extracellular Vesicles

A pivotal finding from EV-sRNA profiling analyses reveals that the composition of sRNAs associated with extracellular vesicles (EVs) distinctly differs from the total sRNA pool [20], hinting at a selective loading process. Investigating this phenomenon, He et al. conducted a proteomic analysis on *Arabidopsis* EVs isolated at high centrifugation speeds and pinpointed several RNA-binding proteins crucial to this selective process [32]. These proteins include the RNA interference (RNAi) component AGO1, DEAD-box RNA helicases (RH11, RH37), and Annexins (ANN1, ANN2). Notably, these proteins co-localize with sRNAs and TET8 in specific sucrose gradient fractions, and their presence persists in TET8-positive exosomes even after trypsin digestion, emphasizing their primary association with exosomal transport.

Further studies by He et al. have established that proteins like AGO1, RH11, and RH37 not only bind selectively to sRNAs found in exosomes but are exclusive to them, compared to sRNAs not associated with EVs [32]. While the *Arabidopsis* genome encodes multiple AGO proteins, only AGO1 is actively secreted via EVs, whereas AGO2, AGO4, and AGO5, along with their specific sRNAs, are absent in the EV fractions. This specificity suggests a critical role for AGO1, RH11, and RH37 in the targeted loading of sRNAs into mainly TET-positive exosomes.

Conversely, Annexins do not exhibit specificity in sRNA binding; however, they are instrumental in stabilizing sRNAs within EVs. This stabilization role is underscored by the observation that sRNA accumulation in EVs is notably diminished in *ann1ann2* mutants [32], indicating the essential function of Annexins in maintaining sRNA integrity within EVs.

### 3.3. Pathogen-to-Host sRNA Trafficking and Its Role in Suppressing Plant Immunity

Plant pathogens are adept at manipulating host cellular mechanisms, as evidenced by their ability to transport small RNAs (sRNAs) into host cells to downregulate host immunity [6,43,48,60] (Figure 1). Notably, the fungal pathogen *B. cinerea* has been shown to deliver sRNAs into cells of *Arabidopsis* and tomato, utilizing the host’s own RNA interference (RNAi) machinery to compromise immune responses [48]. These sRNAs are incorporated into the host AGO1 protein, leading to the suppression of genes crucial for the host’s immune defense, such as MAPKs, cell wall-associated kinases, and those regulating the production of reactive oxygen species.

This phenomenon is not limited to *B. cinerea*; other pathogens, including the fungi *V. dahliae* [62] and *Puccinia striiformis* [63], the oomycete *Hyaloperonospora arabidopsidis* [44], and even the prokaryotic symbiont *Bradyrhizobium japonicum* [64], have similarly been found to exploit this mechanism to transfer sRNAs into host cells. Interestingly, this method of sRNA transfer and gene silencing is consistent across various organisms, from plant and animal fungal pathogens to prokaryotic bacteria.

Beyond the plant world, similar mechanisms are observed in animal systems. For instance, the entomopathogenic fungus (EPF) *Beauveria bassiana* transfers miRNAs to host cells to co-opt the mosquito AGO1 and silence key immune genes like the Toll receptor ligand Spätzle 4 [43].

The use of extracellular vesicles (EVs) for sRNA transfer is also prominent in mammalian systems, where EVs in bodily fluids facilitate intra-organismic sRNA trafficking. This strategy is exploited by various parasites, such as the gastrointestinal nematode *Heligmosomoides polygyrus*, which secretes EVs to deliver miRNAs that modulate inflammation [65].

sRNAs derived from plant pathogens are known to infiltrate host cells and strategically suppress host immunity. For example, the fungal pathogen *B. cinerea* transports sRNAs into *Arabidopsis* and tomato cells, harnessing the host’s RNA interference (RNAi) system to inhibit immune response genes, such as MAPKs, cell wall-associated kinases, and those critical for reactive oxygen species production. This method of manipulating host gene expression via pathogen-derived sRNAs extends beyond *B. cinerea* to include other microbial pathogens like the fungi *V. dahliae* [62] and *Puccinia striiformis* [63], the oomycete *Hyaloperonospora arabidopsidis* [44], and even the symbiotic prokaryote *Bradyrhizobium japonicum* [64], all employing a similar strategy by engaging the host AGO1 protein.

This interaction is not restricted to plant pathogens. For instance, the mosquito fungal pathogen *Beauveria bassiana* [43] transmits an miRNA that targets the mosquito AGO1, silencing critical immune genes such as the Toll receptor ligand Spätzle 4. Moreover, despite lacking RNAi systems, prokaryotes like *Bradyrhizobium* can generate sRNA fragments from tRNAs that bind to plant AGO1, thereby silencing host genes [64].

Future investigations are crucial to determine if plant pathogens similarly use EVs to transport sRNAs to their hosts. Observations have noted the presence of plasma membrane-derived microvesicles at the plant–fungus interface, specifically during interactions between rice roots and the arbuscular mycorrhizal fungus *R. irregularis*, where these vesicles may carry proteins and RNAs essential for signaling across the symbiotic interface [30,31,66].

## 4. Role of Extracellular Vesicles in Plant Defense Mechanisms

The presence of the fungal pathogen *B. cinerea* significantly heightens the production and release of extracellular vesicles (EVs) and small RNAs (sRNAs) in *Arabidopsis* [20], indicating a robust immune response. Similarly, infection by the bacterial pathogen *P. syringae* pv. tomato DC3000, or treatment with the defense hormone salicylic acid (SA), amplifies EV secretion in *Arabidopsis* plants [67,68]. These vesicles have proven their capability to be internalized by fungal pathogens like *B. cinerea* and *Sclerotinia sclerotiorum*, with treatment of *S. sclerotiorum* spores with sunflower-derived EVs leading to inhibited growth, morphological changes, and increased mortality.

*Arabidopsis* EVs are packed with a variety of defense-related proteins, including BAK1-interacting receptor-like kinase 2, Glycine-rich protein 7, RPM1-interacting protein 4, and Suppressor of BIR1-1. These proteins play crucial roles in enhancing pathogen recognition and facilitating immune signaling through the strategic trafficking of signaling molecules.

Additionally, EVs carry key components of the myrosinase-glucosinolate system—glucosinolate transporters like PEN 3 and Glucosinolate transporter 1, and the myrosinase Epithiospecifier modifier 1—which are vital for the plant’s innate immune response against *Pseudomonas syringae* infection and salicylic acid treatment [67,68]. The presence of proteins like PEN1 (Syntaxin-121), Syntaxin-122, and Syntaxin-132 suggests that EVs are also integral in protein transport crucial for immune signaling.

Beyond their defensive role, plant exosomes are instrumental during systemic viral infections. For instance, during Turnip mosaic virus (TuMV) infection in *Nicotiana benthamiana*, multivesicular bodies (MVBs) merge with the plasma membrane to release numerous EVs, as observed through transmission electron microscopy [69]. These EVs, containing TuMV RNA and proteins, suggest a novel pathway for viral release into the extracellular space.

Observations have shown direct uptake of these EVs by fungal cells [1,23,24,70], although the specific mechanisms of EV uptake in plant and fungal cells remain to be elucidated. In mammalian systems, various uptake methods such as phagocytosis, macropinocytosis, and endocytosis have been identified, hinting at possible analogous pathways in plant and fungal cells, involving both clathrin-mediated and clathrin-independent mechanisms. Ongoing research is essential to pinpoint these uptake processes in plant and fungal systems.

## 5. Utilizing Extracellular Vesicles for RNA Interference in Crop Protection

RNA interference (RNAi)-based engineered resistance is achieved by incorporating sequences from pest or pathogen genes into the crop genome. This leads to the production of pathogen-specific double-stranded RNA (dsRNA) or small RNAs (sRNAs), which are pivotal in triggering RNAi. These dsRNAs are processed by the plant’s RNAi machinery, specifically through the action of Dicer enzymes, into siRNA duplexes. These siRNAs are then loaded into AGO proteins, facilitating the targeted degradation of homologous pest or pathogen transcripts, effectively conferring resistance to the genetically modified (GM) crops.

Host-induced gene silencing (HIGS) exploits this mechanism and has shown commercial success in protecting crops against various pests and pathogens [71], from insects like the western corn rootworm [72] to viruses [73]. Despite its efficacy, the widespread adoption of HIGS faces challenges due to the complexity of engineering multiple crops, which requires significant time and resources to tailor the technology to different plant species. Additionally, there are regulatory hurdles associated with genetically modified (GM) crops, including stringent approval processes, public perception issues, and compliance with international trade regulations, all of which can impede the deployment and acceptance of HIGS technology.

**Figure 2:**
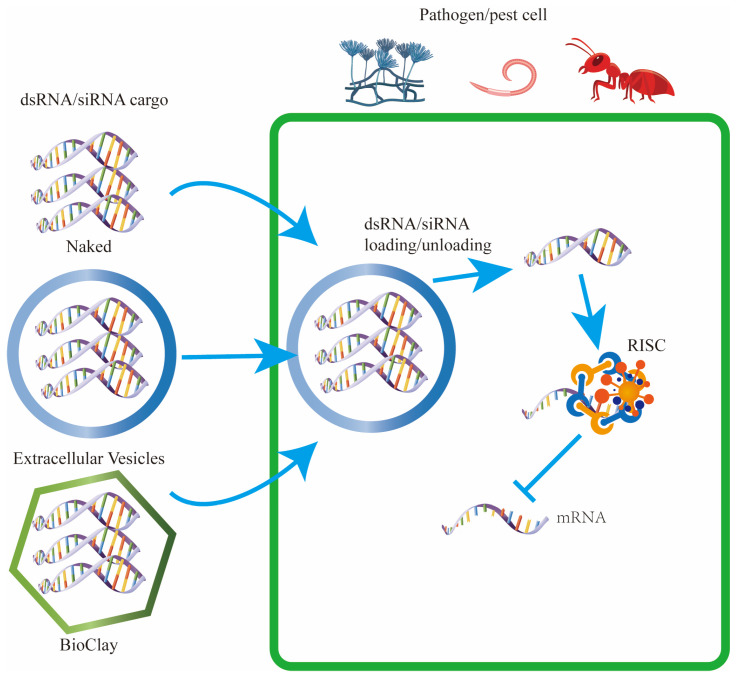
RNAi and Extracellular Vesicles in Crop Protection: Spray-induced gene silencing (SIGS) strategies are employed to deliver RNA molecules, such as dsRNA or siRNA, to crop pathogens and pests including viruses, fungi, nematodes, and insects. Once internalized by the target cells, these RNA molecules trigger RNA interference (RNAi), silencing critical genes and inducing resistance in the host plant. SIGS involves the exogenous application of dsRNA, where nanocarriers like clays, liposomes, or EVs protect the RNAi molecules, improving their stability and facilitating cellular uptake. RISC, RNA-induced silencing complex.

An emerging alternative to HIGS is spray-induced gene silencing (SIGS) (Figure 2), which involves the direct application of dsRNA onto plants to confer protection, bypassing the need for genetic modification of the crop’s genome. Early evidence of SIGS’s effectiveness includes studies from 2001 [74], where foliar application of dsRNA provided resistance against viruses like Pepper mild mottle virus and Tobacco etch virus. Recent advancements have extended SIGS applications to control fungal pathogens such as *B. cinerea* and *F. graminearum*, as well as offering protection against pests like the Colorado potato beetle through dietary exposure to dsRNA [75,76]. Both HIGS and SIGS continue to be pivotal in agricultural research and crop protection strategies, reflecting their importance in modern agronomy and pest management practices.

### 5.1. Mechanisms of Environmental RNA Uptake Across Species

In the model organism *Caenorhabditis elegans*, the process of environmental RNA interference (RNAi) is facilitated by several *systemic RNA interference defective* (SID) genes [77], which are pivotal for RNA transport. Interestingly, homologs of these SID proteins are not present in plants or fungi, indicating species-specific mechanisms of RNA uptake. In eukaryotes, the primary method for internalizing extracellular materials, including RNAs, is through endocytosis. For instance, studies in the red flour beetle (*Tribolium castaneum*) have shown that the disruption of key genes associated with clathrin-dependent endocytosis severely limits the cellular uptake of double-stranded RNA (dsRNA) [78]. Similarly, genetic investigations in *Drosophila melanogaster* S2 cells have identified critical components of the endocytic pathway, such as the clathrin heavy chain and its adaptor proteins [79], underscoring the role of clathrin-mediated endocytosis in dsRNA uptake in this species. This mechanism is also evident in the fungal phytopathogen *Sclerotinia sclerotiorum*, where the application of endocytic inhibitors and RNAi targeting clathrin-mediated endocytosis genes has confirmed their essential role in dsRNA uptake [80].

The efficiency of RNA uptake, however, varies widely among different eukaryotic organisms and cell types. Several aggressive fungal pathogens, including *Botrytis cinerea*, *Sclerotinia sclerotiorum*, *Rhizoctonia solani*, *Aspergillus niger*, and *Verticillium dahliae*, demonstrate a high capacity for environmental dsRNA uptake [81]. Conversely, no RNA uptake has been observed in *Colletotrichum gloeosporioides*, and only minimal uptake occurs in the nonpathogenic fungus *Trichoderma virens*. The oomycete pathogen *Phytophthora infestans* also exhibits limited dsRNA uptake. Notably, the effectiveness of using dsRNAs to target virulence genes correlates with the pathogen’s RNA uptake efficiency [81], playing a critical role in the success of spray-induced gene silencing (SIGS) for crop protection (Figure 2).

### 5.2. Utilizing Extracellular Vesicles and Nanoparticles for RNA Interference in Agricultural Applications

For spray-induced gene silencing (SIGS) to achieve its full potential, overcoming the challenges associated with enhancing dsRNA uptake and ensuring its efficient distribution to achieve optimal dosages is crucial. Extracellular vesicles (EVs), known for their role in cross-kingdom RNA interference and plant defense mechanisms, present a promising avenue for facilitating RNAi-mediated crop protection. The successful application of nanovesicles as RNAi delivery vehicles in mammalian systems offers valuable insights and precedents [65,82]. These can be adapted and applied to plant systems, potentially revolutionizing how dsRNA is delivered for crop protection through SIGS, leveraging lessons learned from medical research to enhance the efficacy of agricultural applications.

The strategic use of RNA interference (RNAi) in crop protection greatly benefits from the precise timing and localization of RNAi trigger molecules. To address this, various carriers have been developed to protect dsRNA at the point of application and improve its absorption and distribution to both local and distal plant tissues. Drawing on successes from medical therapeutics, researchers are increasingly turning to both synthetic and natural nanocarriers to solve these delivery challenges.

One notable success is BioClay (Figure 2), an innovative formulation combining layered double hydroxide (LDH) particles with target-specific dsRNA [83,84]. This nanocomposite has demonstrated the ability to prolong the protective effects against plant viruses from several days to weeks through controlled dsRNA release initiated by the dissolution of the LDH under acidic conditions. This method of SIGS does not require genetic modification and induces systemic plant resistance.

Additionally, carbon quantum dots have emerged as effective carriers, facilitating the systemic RNAi in agricultural pests like the rice striped stem borer when applied topically [85,86]. Another breakthrough has been the development of a nanotube-based platform that delivers siRNAs directly into plant cells, enhancing gene silencing efficiency by protecting the siRNA from degradation by nucleases [87].

The extensive research on using liposomes and EVs for RNAi delivery in medical settings is starting to influence agricultural practices. Recent applications include the successful use of liposomes to deliver dsRNA to pests like the Neotropical stink bug *Euschistus heros* [88] and the Queensland fruit fly *Bactrocera tryoni* [89]. This success prompts the question of whether similar strategies using EVs or organic nanovesicles could be adapted for SIGS to enhance RNAi efficacy against agricultural pests and pathogens.

Furthermore, recent findings suggest that siRNAs from fungal pathogens like *F. graminearum*, intended for host-induced gene silencing (HIGS) but not SIGS, are present in *Arabidopsis* EVs [90]. This observation raises the possibility that pre-loading dsRNA or siRNA onto EVs before their application might provide additional protective and delivery advantages not inherently available through the plant’s own systems.

### 5.3. Enhancing RNAi Delivery in Plant Protection Using Extracellular Vesicles

The utilization of extracellular vesicles (EVs) offers substantial advantages for the delivery of dsRNA to plant cells, particularly in combating viruses where cellular uptake of dsRNA is crucial. Despite the plant cell wall typically having a pore size of around 5 nm—smaller than most EVs—successful delivery has been observed. This suggests that either the EVs exhibit enough structural plasticity to pass through these pores, or that the plant cell walls themselves adapt to facilitate the passage of EVs and their RNA contents (Figure 2) [66].

In contrast to synthetic agents like carbon nanotubes [91], which can bypass pore size restrictions due to their high aspect ratio but may persist in the environment, EVs are likely to degrade more rapidly. This biodegradability, coupled with their biocompatibility, is a vital aspect for gaining regulatory approval for use in crop protection.

The specificity with which functionalized liposomes or natural EVs can target payloads to particular cell types is invaluable. This is especially relevant for combating plant viruses that exhibit tissue tropism, like certain begomoviruses known to infect phloem cells exclusively. Enhancing dsRNA delivery to these specific cells could significantly increase the efficacy of RNAi-based treatments against such viruses.

Moreover, the localized delivery of dsRNA could also be crucial for addressing insect pests that feed on phloem sap or fungi that primarily target specific plant tissues like roots, potentially increasing the uptake and effectiveness of RNAi strategies. As spray-induced gene silencing (SIGS) technologies evolve, the unique delivery capabilities of EVs are expected to highlight additional protective benefits.

This shift towards utilizing targeted, biodegradable delivery systems like EVs marks a significant move from traditional methods towards more sustainable, non-GM crop protection strategies. Nonetheless, the potential for EVs to affect non-target species inadvertently remains a concern that necessitates thorough investigation on a case-by-case basis to ensure environmental safety.

### 5.4. Extracellular Vesicles as Versatile Carriers for Diverse Beneficial Cargos

Extracellular vesicles (EVs), along with synthetic vesicles, have shown significant potential beyond just the delivery of nucleic acids. They can also transport a range of other beneficial molecules, particularly in therapeutic contexts. For instance, EVs have been used to carry plant-derived anticancer bioactives like celastrol and curcumin, as well as gold nanoparticles that enhance imaging capabilities [17,92]. The ability of EVs to protect these cargos from environmental factors—such as degradation during oral delivery—and efficiently transport them to specific tissues and cells highlights their utility.

An additional benefit of using EVs involves minimizing the chances of adverse interactions between the cargo and other pharmaceuticals. Unlike liposomes, EVs have the capability to cross the blood-brain barrier [93], making them particularly valuable for delivering drugs to the central nervous system.

EVs can encapsulate both hydrophilic molecules within their aqueous interiors and hydrophobic molecules within their lipid bilayers. However, the methods for loading these cargos and the efficiency of loading can vary significantly depending on the EV source and the nature of the cargo. Leveraging the natural cellular processes for selective sorting and packaging into EVs could enhance both the loading efficiency and the delivery precision of these active compounds. This technology holds promise for agricultural applications as well, such as delivering nutrients directly to plant tissues or providing targeted distribution of pesticides.

The use of delivery agents like gold and silica nanoparticles has been explored for these purposes, but EVs might offer superior delivery efficiency and cell-type targeting capabilities. Additionally, the potential for combining multiple types of beneficial cargos, such as dsRNA with other agents, within a single EV, mirrors the multifunctionality seen in drug-loaded liposomes. This approach could reduce the environmental footprint associated with widespread agricultural applications, offering a more sustainable solution for managing broadacre crops.

## 6. Conclusions: The Dynamic Role of Extracellular Vesicles in Plant–Pathogen Interactions

Our discussion has highlighted the significant role of extracellular vesicles (EVs) in facilitating communication across kingdoms, particularly between plants and their microbial pathogens. Plants have been shown to harness EVs to transport defensive cargos, including small RNAs (sRNAs), to combat infections. These plant-derived EVs actively deliver sRNAs into pathogens, thereby orchestrating cross-kingdom RNA interference (RNAi).

In mammalian systems, EVs carry not only sRNAs but also mRNA and long noncoding RNAs (lncRNAs), which play crucial roles in cell-to-cell communication [65]. It remains to be explored whether similar RNA types are also exchanged between plant and pathogen cells through EVs. Pathogens, on their part, may exploit EVs to transport virulence factors such as proteins and RNAs into plant cells, enhancing their infectious capabilities.

Despite the growing body of research on plant and mammalian EVs, studies focusing on microbial EVs, especially those derived from diverse pathogens, are relatively sparse. Typically, microbial EVs are isolated from cultured media under in vitro conditions; however, exploring EVs extracted from extracellular fluids of infected plants could provide deeper insights into their roles during infection.

Furthermore, in animal models, various RNA-binding proteins have been identified as key players in miRNA sorting into specific EV subtypes. Similarly, in plants, proteins such as AGO1 and RNA helicases are integral for the selective loading of sRNAs into EVs. The discovery of different molecule families and pathways that contribute to exosome formation and secretion highlights the complexity and diversity of EVs, suggesting the existence of distinct subtypes among them.

Advancements in immunoaffinity isolation techniques, such as the development of multiplex bead-based immunoaffinity systems, have enabled the precise capture of specific EV subtypes using antibodies against known EV markers like tetraspanins CD9, CD81, and CD63. This method holds promise for isolating plant exosomes directly from interaction interfaces for detailed analysis of their RNA, protein, and metabolite contents. Although EV-based technologies do not directly modify the genetic makeup of crops, they may still encounter similar scrutiny because of their molecular characteristics and the perception that they resemble genetically modified organisms (GMOs). To facilitate their adoption in agriculture, it is essential to implement various measures, such as conducting comprehensive safety assessments, engaging with regulatory bodies early in the development process, and collaborating with both public and private stakeholders. These efforts are crucial to ensure that the technologies align with societal values and expectations and to effectively communicate with the public.

The Minimal Information for Studies of Extracellular Vesicles (MISEV) guidelines provide a comprehensive framework to standardize the characterization and reporting of EV research, ensuring reproducibility and reliability in the field [94]. Rigorous characterization of EVs’ purity and the methods of isolation are crucial. For plant applications, it is essential to use standardized and validated methods to ensure the EVs are free from contaminants that could affect the plant systems. Physical, biochemical, and functional characterization of EVs is required. MISEV guidelines suggest using multiple complementary techniques such as electron microscopy, nanoparticle tracking analysis, and flow cytometry to thoroughly characterize EVs. This ensures a clear understanding of their composition and functionality. Detailed reporting of experimental protocols, including EV source, isolation methods, and characterization techniques, is mandated by MISEV guidelines [94]. This could lead to the development of innovative strategies to deliver protective agents such as RNAs and pave the way for the development of sustainable solutions to enhance plant health and agricultural productivity.

## Figures and Tables

**Table 1:** Typical experimentally validated studies conducted on extracellular vesicles and cross kingdom RNAi.

Host Species	Parasite/Mutualist Microbe	EV Cargo with Biological Activity	Reference
*Arabidopsis thaliana*	*Botrytis cinerea*	21 nt sRNAs Bc-siR3.1 Bc-siR3.2 Bc-siR5	[48]
*Solanum lycopersicum*	*Botrytis cinerea*	21 nt sRNA Bc-siR5	[48]
*Arabidopsis thaliana*	*Botrytis cinerea*	Bc-siR37	[49]
*Triticum aestivum*	*Puccinia striiformis* f. sp. *tritici*	miRNA-like (milR1)	[50]
*Arabidopsis thaliana*	*Cuscuta campestris*	22 nt miRNAs e.g., miR393	[51]
*Solanum lycopersicum*	*Fusarium oxysporum* f. sp. *lycopersici*	23 nt miRNA-like Fol-milR1	[52]
*Triticum aestivum*	*Puccinia striiformis* f.sp. *tritici*	17 20–21 nt sRNAs	[53]
*Malus* × *domestica*	*Valsa mali*	miRNA-like Vm-milR1	[54]
*Oryza sativa*	*Xanthomonas oryzae* pv. *oryzicola*	Xosr001	[55]
*Gossypium hirsutum*	*Verticillium dahliae*	miR166/miR159	[56]
*Hordeum vulgare*	*Blumeria hordei*		[9]
*Arabidopsis thaliana*	*Verticillium dahliae*	miR166/miR159	[57]

## Data Availability

No new data were created or analyzed in this study. Data sharing is not applicable to this article.
